# Transcriptomic Analysis Following Artificial Selection for Grasshopper Size

**DOI:** 10.3390/insects11030176

**Published:** 2020-03-10

**Authors:** Shuang Li, Dong-Nan Cui, Hidayat Ullah, Jun Chen, Shao-Fang Liu, Douglas W. Whitman, Ze-Hua Zhang, Xiong-Bing Tu

**Affiliations:** 1 State Key Laboratory for Biology of Plant Diseases and Insect Pests, Institute of Plant Protection, Chinese Academy of Agricultural Sciences, Beijing 100193, China; sclishuang61@163.com (S.L.); Cuidongnan88@163.com (D.-N.C.); shabkadar@yahoo.com (H.U.); cjhp2014@163.com (J.C.); liushaofanghnpy@163.com (S.-F.L.); 2 Department of Agriculture, The University of Swabi, Anbar 23561, Khyber Pakhtunkhwa, Pakistan; 3 School of Chemistry, Biology and Materials Science, East China University of Technology, Nanchang-330013, China; 4 School of Biological Sciences, Illinois State University, Normal, IL 61761, USA

**Keywords:** transcriptome, PI3K-Akt, phenylalanine metabolism, Mtor, ECM–receptor interaction, DEGs, *Romalea microptera*, body size, rapid evolution, genetic correlation

## Abstract

We analyzed the transcriptomes of *Romalea microptera* grasshoppers after 8 years of artificial selection for either long or short thoraces. Evolution proceeded rapidly during the experiment, with a 13.3% increase and a 32.2% decrease in mean pronotum lengths (sexes combined) in the up- and down-selected colonies, respectively, after only 11 generations. At least 16 additional traits also diverged between the two colonies during the selection experiment. Transcriptomic analysis identified 693 differentially expressed genes, with 386 upregulated and 307 downregulated (55.7% vs. 44.3%), including cellular process, metabolic process, binding, general function prediction only, and signal transduction mechanisms. Many of the differentially expressed genes (DEGs) are known to influence animal body size.

## 1. Introduction

Artificial selection is a valuable tool for testing and understanding natural selection, evolution, and the genetics underlying these processes [1,2,3]. Indeed, knowledge of artificial selection was foundational for the cognitive development of Darwin’s evolutionary theory [4], as shown by the fact that he devoted his first chapter in *On the Origin of Species by Natural Selection* to domestication and selective breeding [5]. Today, “artificial selection” generally refers to active selection (culling) by humans, whereas “experimental evolution” refers to experiments where humans do not actively select individuals for further breeding, but instead where populations are placed into specific environmental/experimental conditions selected by humans, and thereafter allowed to evolve on their own over successive generations [6,7]. Domestication is a type of long-term artificial selection. All three represent selection and evolution directed by humans.

The great value of artificial selection is that it represents applied evolution in real time. Given some background genetic variation in the experimental population, virtually any trait can be modified by artificial selection [8,9,10]. Like natural selection, artificial selection can alter allele frequencies in populations, resulting in altered, inherited phenotypes [11,12,13,14]. Artificial selection, domestication, and experimental evolution can produce substantial phenotypic change [15,16,17] and can be stunningly fast, with clear divergence sometimes in as little as three to five generations [18,19,20,21,22,23,24,25]. In fact, evolution by artificial selection can be instantaneous when an experimenter removes from a given population all individuals possessing a phenotype determined by a dominant allele. As such, artificial selection allows the rapid testing of evolutionary hypotheses and the elucidation of the processes and molecular mechanisms underlying evolution and trait expression.

Today, modern molecular biology allows us to quickly know the genetic changes resulting from artificial selection—genetic changes that alter phenotypes. When combined with molecular and other analysis, comprehensive artificial selection experiments can help reveal the entire multilevel, multisystem sequence of events that occurs during and as a consequence of evolution, from selection to changes in genomes → transcripts → proteins → metabolism → cellular physiology → system physiology → development, and on to morphology, life-history, and behavior [6,7]. Additional research can explore the fitness and ecological consequences of phenotypic changes produced by artificial selection. Such broad and deep understanding has been achieved in a number of model study organisms, most notably in *Drosophila* [26,27,28,29,30].

One surprising finding of artificial selection research is that focused selection on a single trait usually also alters numerous other traits, some of which were previously unknown and unexpected to be related to the focus-trait [2,6,10,24,25,31,32]. Examples of these are observed in silver foxes selected only for tameness (a behavioral trait), regarding the evolution of light fur color, spotted coats, droopy ears, short snouts, narrow skulls, shorter, curled tails, under- and over-bites, shorter legs, tail wagging, loss of musky smell, and larger litter size (mostly morphological traits) [33,34]. Such “side-effects” of artificial selection were recognized by Darwin [5] and can be caused by pleiotropy, linkage disequilibrium, genetic drift, developmental, phenotypic, and functional correlations, and (in long-running or high-population experiments) mutation [3,14].

In this paper, we compare transcriptomes of grasshoppers after laboratory selection for long vs. short pronotum length, in order to understand molecular mechanisms underlying evolution and genes and pathways that determine body size. Transcriptomic analysis following artificial selection allows us to link phenotypic change to gene expression, and, thus can help identify genes and pathways that contribute to selection-caused phenotypic changes. We hypothesize that many of these transcriptional differences represent genes active in constructing and directing divergent phenotypes resulting from up- vs. down-artificial selection on body size.

An unanticipated discovery from early transcriptomic analysis was the sometimes very large number of transcriptional changes that resulted from artificial selection, including many influencing unexpected proteins/systems/traits. Although some of these undoubtedly represent nonadaptive hitchhiking, genetic drift, and mutation, many represent evolved pleiotropy. As such, transcriptomic analysis has helped scientists to understand the polygenic nature of complex quantitative traits, discover new pleiotropic relationships, assign functions to previously unstudied or unannotated genes, understand the complex and extensive nature of pleiotropy, and to observe genome evolution and epigenetics in real time, thus further revealing how biological systems work [14,34,35]. 

The subject of our study, body size, is an excellent focus trait for artificial selection and evolutionary studies, because, for nearly all species, body size is highly variable, easily measured and selected, polygenic and correlated with numerous other traits, has high heritability, and is fundamental to nearly all other traits. Size influences physiology, ecology, life history, behavior, performance, fitness, and evolution [36,37]. This current paper is part of a long-term study of body size in Orthoptera in our laboratories [36,37,38,39,40,41,42,43,44,45,46,47,48].

## 2. Materials and Methods 

### 2.1. Ethics Statement

*Romalea microptera* (Beauvois) were fed in the biological science laboratory in Illinois State University. *R. microptera* is an agricultural pest and is not on the ‘List of Protected Animals in America‘. No permits were required for the field studies.

### 2.2. The Insect 

The eastern lubber grasshopper, *R. microptera* (Beauvois) (Orthoptera; Romaleidae) ranges across the southeastern USA [49,50]. It is a model study organism due to its large size (up to 7 cm long and 21 g), long life, calm demeanor, flightlessness, interesting biology, and ease of laboratory culture [51]. This species, and Orthoptera in general, exhibit considerable inter-and intrapopulational variation in body size in nature, and this variation has both genetic and environmental origins [37,41,43,46].

### 2.3. The Artificial Selection Experiment

We selected for long- vs. short-pronotum length for 8 years (11 generations) in two laboratory colonies of *R. microptera* that originated from 80 adults collected from the field at Shark Valley, Everglades National Park, Florida, USA in 2005 [43,44]. These wild insects were transported to Illinois State University and maintained and allowed to mate at will for one generation in a large communal cage [51], producing ~80 egg pods. The resulting hatchlings gave rise to ~230 adults. From this single population (referred to as the Parent Lab Population), we selected the 30 adults with the longest pronotum length and the 30 adults with the shortest pronotum length (15 of each sex/treatment) to initiate our artificial selection experiment. These animals formed Generation 1 of the Large Colony and the Small Colony, respectively. For the next 8 years we maintained these two colonies in separate cages, while always selecting the largest ~15% and smallest ~15% of individuals of each generation for further breeding. Throughout the experiment, we used Mitutoyo Inc. Model CD-6 digital calipers to measure the lengths of their thoraces along the dorsal mid-line. Each individual was measured by two different researchers. If the two measurements were not identical, the individual was remeasured until a consensus was found. 

### 2.4. RNA Extraction and Sequencing

Molecular analysis was undertaken by the State Key Laboratory for Biology of Plant Diseases and Insect Pests, Institute of Plant Protection, Chinese Academy of Agricultural Sciences. Total RNA was isolated from the whole body of five female adults of the Large Colony and from the same number and sex of Small Colony individuals with Trizol (Invitrogen) as per the manufacturer’s protocol. Environmental effects on transcription were minimized by rearing the two colonies side-by-side under identical conditions. Quality and quantity of RNA samples were determined with a Thermo Scientific NanoDrop^TM^ ND-1000 spectrophotometer (Implen, CA, USA). Total RNA was treated with RNase-free Dnase I (New England BioLabs, USA) to remove contaminants of residual DNA. The first complementary DNA (cDNA) strand was synthesized using random hexamer primers and M-MuLV Reverse Transcriptase (Rnase H^-^). The double-stranded cDNA fragments were processed by end repair using T4 DNA polymerase, Klenow DNA polymerase, and T4 polynucleotide kinase (NEB, USA), followed by a single adenine base addition using Klenow 39 to 59 exo-polymerase, and was concluded by ligation with Illumina’s adaptor. The products were purified using a QIAquick PCR extraction kit (Qiagen) and enriched by PCR amplification. The library products were sequenced on an Illumina Hiseq 4000 platform (Illumina, San Diego, CA, USA) and 150 bp paired-end reads were subsequently generated by Novogene Corporation (Novogene, Tianjin, China) as per the protocol [52].

### 2.5. Bioinformatics Analyses

In order to obtain clean and high-quality data, clean data (clean reads) were obtained by removing reads containing adapter, reads containing ploy-N and the low-quality reads from the raw data. At the same time, Q20, Q30, and the GC content and sequence duplication level of clean data were calculated. Later on, we used Trinity to assemble the clean data and took the transcriptome from Trinity assembled as the reference sequence (Ref), mapping the clean reads of each sample on the Ref. The longest transcript of each gene was taken as unigene for further analysis [53,54]. All unigenes assembled were compared respectively with the nonredundant protein database (NR) of the National Center for Biotechnology Information (NCBI), nonredundant nucleotide sequence (NT) of NCBI database, UniProt/Swiss-Prot, Gene Ontology (GO), and the Keeper of the Grove (KOG) database. Similarly, the Gene-list enrichment was completed using KOBAS 3.0 [53,54].

DEG analysis of two samples was performed using the DEGseq R package [53,54]. *p* value was adjusted using *p* value [54,55]. *p* value ≤ 0.05 and |log_2_(fold change)| ≥ 1 was set as the threshold for significantly differential expression. To enrich the identified genes, especially the differential genes involved in the canonical pathway, the Kyoto Encyclopedia of Genes and Genomes database and GO enrichment analysis were carried out as described previously through cluster Profiler R Package [53,54,55].

### 2.6. Accession Number(s)

*R. microptera* transcriptome datasets are available at NCBI, under project No. PRJNA392010 with accession number SRP110722, and at SRA with accession number SRS2324007. All other data are contained in the manuscript and in the Supplementary Materials.

## 3. Results

### 3.1. Adult Pronotum Length

During our study, adult pronotum length responded to both up- and down-selection (Table 1). After 11 generations, mean male and female pronotum length had increased in the Large Colony by 14.8% and 11.8%, respectively, and decreased in the Small Colony by 31.3% and 33.0%, respectively (Table 1). Interestingly, although the only selection criterion was pronotum length, at least 16 additional traits had changed by the end of the selection experiment, including development rate, number of molts, number and size of ovarioles and eggs, behavior, body color, and numerous other morphological traits. By year 8, adult body mass and volume in the Large Colony was almost double that of the Small Colony. A comprehensive description of the methods and results of the selection experiment, emphasizing these phenotypic changes, will be published in a separate paper.

### 3.2. Unigenes and Magnitude of Expression Differences

Following de novo assembly [55], a total of 120,267 unigenes were generated from both samples with a total length of 82,796,396 bp and the N50 of 1149 bp (Figure S1). Among these, 693 genes were differentially expressed in samples from the Large Colony vs. the Small Colony (*p* < 0.05), with 386 upregulated and 307 downregulated (55.7% vs. 44.3%) (Tables S1 and S2). Overall, the magnitude of expression differences tended to be high, with 335 of 693 DEG (48.3%) exhibiting at least a 5-fold difference in expression level. However, up- and downregulated genes tended to differ in their levels of expression (Table 2). More upregulated genes exhibited >2-fold expression vs. downregulated genes (362 vs. 267, respectively). However, downregulated genes predominated (40 vs. 24) when DEG was less than 2-fold (Table 2).

### 3.3. Functional Annotation of Unigenes

**Figure 1:**
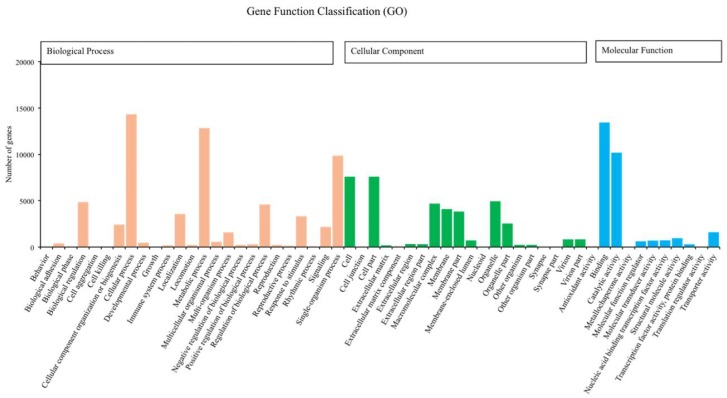
Number of unigenes of *Romalea microptera* explained and classified by Gene Ontology (GO) data library in different functional groups within the categories of biological process, cellular component, and molecular function.

**Figure 2:**
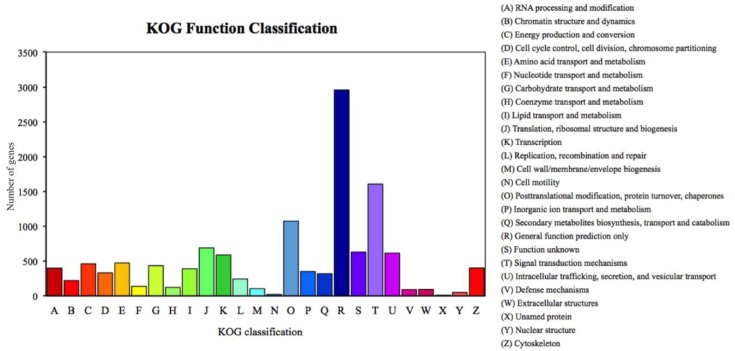
Percent of *Romalea microptera*’s unigenes in each functional category for expressivity and dominancy, explained and classified by the Keeper of the Grove (KOG) database.

We annotated 39,395 of 120,267 unigenes via seven databases: NR (29,217) > GO (24,742) > PFAM (24,622) > SwissProt (17,521) > KOG (11,500) > KO (9,207) > NT (8,007). Among the most dominant functional classifications as explained by public database GO, were biological process designated as ‘cell process‘, ‘metabolic process‘ and ‘single organism process‘, followed by molecular function with terms ‘binding‘ and ‘catalytic activity‘, and cellular component with terms ‘cell‘ and ‘cell part‘ (Figure 1). In the category of biological process, a total of 12,826 genes were absolutely related with the metabolic process. On the other hand, the KOG functional classifications respectively explained ‘general function prediction only (R)’, ‘signal transduction mechanisms (T)’, ‘post-translational modification protein turnover chaperones (O)’ and ‘translation ribosomal structure and biogenesis (J)’ as the most dominated functions (Figure 2).

### 3.4. Functional Characterization

To understand the molecular changes resulting from size-selection in *R. microptera*, we used clusterProfiler R Package to analyze the DEGs enriched from GO and KEGG databases. The GO database revealed that seven terms were substantially enriched (*p* < 0.05), including DNA metabolic process (49; 25.7%), DNA replication (31; 16.2%), RNA-dependent DNA replication (27; 14.1%), DNA integration (16; 8.4%) under the category of biological processes and nucleotidyltransferase activity (32; 16.8%), DNA polymerase activity (30; 15.7%) and RNA-directed DNA polymerase activity (27; 14.1%) within the category of molecular function (Table 3). To test for genes that might influence size-regulation in *R. microptera*, we used KOBAS 3.0 to find statistically enriched DEGs of downregulated and upregulated features in KEGG pathways [53]. The 307 downregulated DEGs were mapped to 30 pathways in the KEGG database. Among them, only 10 pathways were substantially enriched (*p* < 0.05) involving single genes except for Huntington’s disease, which had two genes (Table 4). Besides, Huntington’s disease, phenylalanine metabolism, and other glycan degradation were among the top contributing pathway terms. Similarly, 386 upregulated DEGs were mapped to a total of 21 pathways, which were substantially richer (Table 5). Furthermore, the number of genes participating in most of these upregulated pathways was higher than those of DEGs negatively expressed. Fat digestion and absorption (2), glycerolipid metabolism (2), metabolic pathways (5), and ECM–receptor interaction (2) were among the highly represented pathway terms.

## 4. Discussion

In our study, we selected for either long or short pronotum length in a grasshopper. After 8 years (11 generations) of artificial selection in the laboratory, pronotum length had increased ~13.3% in the population selected for long thoraces and decreased ~32.2% in the population selected for short thoraces (sexes combined) (Table 1). In addition, although we had selected for only a single trait, at least 16 other traits had diverged between the two populations, including overall body size and mass: adults from the Large Colony had become nearly twice the mass of those from the Small Colony (unpublished). These results suggest that artificial selection on a single trait influenced numerous genes, causing profound changes in multiple physiological processes across multiple systems, and induced multiple phenotypic changes. Multitrait changes are common in selection experiments and are attributable to pleiotropy, linkage disequilibrium, genetic drift, and (in long-term or high-population experiments) mutation [7,14,34].

Evolution proceeded rapidly during our experiment, altering numerous traits in only 11 generations. Indeed, both natural and artificial selection can be stunningly fast [23,25,56,57]. Our experiment employed relatively low population sizes. Generation 1 started with only 15 individuals of each sex/treatment, and subsequent generations started with between 20 and ~65 breeding individuals/treatment, after removing ~85% of individuals with inappropriate pronotum lengths. Hence, we employed a moderate level of selection, with ~15% of individuals selected for continued breeding during each generation. We do not know how our population size and level of selection influenced our results. Researchers have debated the effects of different population sizes and selection intensities on artificial selection, with some suggesting that small population size quickly reduces genetic variation and that intense selection acts as a bottleneck, eliminating important background genetic variation and variation for the focus trait, as well as lowering overall colony fitness [1,7,14].

In this experiment, we sought to characterize the transcriptomic responses to artificial selection as a first step in identifying putative genes and biochemical pathways involved in evolution and regulation of body size and other altered traits. We assume that the divergent phenotypes resulting from our selection experiment derived from altered genotypes. To begin to understand these possible genetic changes, and to identify possible biological pathways active in size regulation, we analyzed, annotated, and compared the transcriptomes of the two colonies. We found comparatively higher ratios for upregulated vs. downregulated DEGs in both groups. Similarly, log_2_ (fold change) at level 2–3 and >5 represented maximum contribution for DEGs. Among DEGs were those active in controlling metabolic process, cell process, and binding, including the phosphatidylinositol-3-kinase/protein kinase B (PI3K-Akt) pathway previously reported in other insects for cellular responses such as cell proliferation, apoptosis, DNA repair, and protein synthesis [58]—all related in some way to growth and development. KOG function analysis described maximum contribution of signal transduction mechanisms, post-translational modification, protein turnover and chaperones coupled with general function prediction for size variation in insects. Similarly, within the biological pathways, the active terms identified by the GO database associated with body size of *R. microptera* were DNA metabolic process and DNA replication, besides the other pathways, such as biological processes and cellular components. Laminin and collagen related with the formation of basement membrane are important genes of the PI3K pathway. In our results, collagen and laminin exhibited an upregulated expression pattern in the Large Colony (Table S1, S2). These collagens are reported to be structural proteins involved in the synthesis of a variety of extracellular matrices in animals [59,60]. For example, in nematodes, such as *Caenorhabditis elegans*, the role of collagen is associated with the formation of basement membrane and cuticle, and cuticle collagens can act as regulators of body size [61,62]. Laminins, involved in PI3K-Akt pathway, are a major component of the basement membrane and contribute in most biological and ecological functions [63,64]. As such, laminin and collagen might be involved in the body size regulation of lubber grasshoppers.

Leptin is another fat-cell-specific hormone influencing development, growth, metabolism and reproduction by binding to the leptin receptor (lepr) whereas leptin receptor gene-related protein negatively regulates leptin-receptor cell surface expression and thus decreases response to leptin [65,66]. In mammals, leptin receptor gene-related protein influences growth hormone signaling that regulates body size and metabolism [67]. In this study, we found that the leptin receptor gene-related protein in *R. microptera* has no such significant contribution in body size at the transcriptomic level. We found that ECM–receptor interaction had a positive role in upregulated DEGs of both samples. For example, Spondin-1 is a cell adhesion protein usually attached to the sensory neuron cells and outgrowth of neuritis that encode a secreted basement membrane molecule similar in function to F-spondin of vertebrate [68,69]. In our study, the population selected for long-thorax (Large Colony) exhibited an upregulated expression pattern for spondin-1, which could be involved in insect body size regulation. Similarly, we identified three upregulated carboxylesterases in the Large Colony. Carboxylesterase has several functions, including regulating development, metabolic detoxification of insecticides and exogenous substances, hormone degradation, and neurogenesis [70].

As discussed, above, numerous phenotypic traits and transcripts appeared to change during our artificial selection experiment. At this time, we cannot assign a direct cause-and-effect relationship between any specific altered transcription product and any specific altered trait. However, this paper is a necessary first step toward that elucidation.

## 5. Conclusions

Our experiment confirms that artificial selection on a single morphological trait (pronotum length) in small populations can quickly alter numerous transcripts and phenotypic traits, including nonmorphological traits, such as behavior and life-history traits. As such, our study demonstrates rapid genetic and phenotypic evolution, apparently accompanied by strong genetic correlations during artificial selection. Only 11 generations of selection produced evolution in at least 17 phenotypic traits and 693 DEGs, including many known to influence body size in animals, like cuticle protein 34, cuticle protein 8, structural constituent of cuticle, insect cuticle protein, and pro-resilin. We hypothesize that pleiotropy, linkage disequilibrium, and genetic drift during artificial selection in small populations induced these observed genomic and phenotypic changes.

## Figures and Tables

**Table 1:** Change in mean pronotum length of *Romalea microptera* grasshoppers during 8 years (11 generations) of artificial selection in the laboratory for either long- or short-pronotum length. See Section 2 for description of selection experiment and populations.

Population	Year	Male		Female	
		*n*	Pronotum Length (mm) Mean ± SD	*n*	Pronotum Length (mm) Mean ± SD
Wild	2005	20	17.8 ± 1.09	20	21.1 ± 2.10
Parent (Lab)	2006	50	18.2 ± 0.97	50	21.2 ± 1.33
Large Colony Generation 11	2014	41	20.9 ± 1.17	46	23.7 ± 1.39
Small Colony Generation 11	2014	31	12.5 ± 0.75	26	14.2 ± 0.70

**Table 2:** Fold-change distribution of DEGs performing Illumina sequencing and de novo assembly for two colonies of *Romalea microptera*.

Log_2_ (Fold Change)		1–2	2–3	3–4	4–5	>5	Total
LG vs. SG	Downregulated	40	52	42	40	133	307
	Upregulated	24	63	46	51	202	386
Total		64	115	88	91	335	693

**Table 3:** Substantially enriched GO terms of DEGs.

Term type	Description	*p*-Value	DEGs Number	Percentage (%)
Biological process	DNA integration	0.0107	16	8.38
Biological process	DNA metabolic process	0.00174	49	25.65
Biological process	RNA-dependent DNA replication	0.00435	27	14.14
Biological process	DNA replication	0.0195	31	16.23
Molecular function	DNA polymerase activity	0.00174	30	15.71
Molecular function	RNA-directed DNA polymerase activity	0.00435	27	14.14
Molecular function	Nucleotidyltransferase activity	0.0216	32	16.75

**Table 4:** Number of substantially enriched pathways along with number of genes of downregulated DEGs computed by Kyoto Encyclopedia of Genes and Genomes (KEGG) pathway analysis.

S. No.	Pathway Term	*p*-Value	Gene Number
1	Huntington’s disease	0.0180808	2
2	Phenylalanine metabolism	0.0188286	1
3	Other glycan degradation	0.0198644	1
4	Histidine metabolism	0.026057	1
5	Tyrosine metabolism	0.037311	1
6	Alanine, aspartate, and glutamate metabolism	0.037311	1
7	Tryptophan metabolism	0.0423843	1
8	Glycine, serine, and threonine metabolism	0.0423843	1
9	Cysteine and methionine metabolism	0.0474316	1
10	Hedgehog signaling pathway	0.0494433	1

**Table 5:** Number of substantially enriched pathways along with number of genes of upregulated DEGs computed by KEGG pathway analysis.

S. No.	Pathway Term	*p*-Value	Gene Number
1	Fat digestion and absorption	0.00031	2
2	Glycerolipid metabolism	0.000625	2
3	Metabolic pathways	0.000779	5
4	ECM–receptor interaction	0.00118	2
5	Small cell lung cancer	0.001293	2
6	Amoebiasis	0.001732	2
7	Cell adhesion molecules (CAMs)	0.003595	2
8	mTOR signaling pathway	0.003984	2
9	Focal adhesion	0.006768	2
10	Pantothenate and CoA biosynthesis	0.011402	1
11	Steroid biosynthesis	0.012595	1
12	PI3K-Akt signaling pathway	0.018149	2
13	Galactose metabolism	0.019132	1
14	beta-Alanine metabolism	0.019132	1
15	Pathways in cancer	0.023941	2
16	ABC transporters	0.027392	1
17	Drug metabolism—other enzymes	0.027979	1
18	Carbohydrate digestion and absorption	0.027979	1
19	Retinol metabolism	0.039076	1
20	Bile secretion	0.042555	1
21	Complement and coagulation cascades	0.047174	1

## Supplementary Materials

The following are available online at https://www.mdpi.com/2075-4450/11/3/176/s1, Figure S1: Unigene length distribution of transcriptome assembly in Romalea microptera, Table S1: Down Regulated Genes; Table S2: Up Regulated Genes

## Abbreviations

DEGs	Differentially Expressed Genes
GO	Gene Ontology
KEGG	Kyoto Encyclopedia of Genes and Genomes
SC	Small colony
LG	Large colony
NR	NCBI nonredundant protein sequences
Pfam	Protein family
KOG	Keeper of the Grove database
KO	KEGG Ortholog database
Nt	NCBI nonredundant nucleotide sequences
Swiss-Prot	Manually annotated and reviewed protein sequence database
CAMs	Cell adhesion molecules
cDNA	Complementary DNA
DNA	Deoxyribonucleic acid
RNA	Ribonucleic acid
NCBI	National Center for Biotechnology Information
