# Supplementary figures and images for: Transcriptomic Analysis Following Artificial Selection for Grasshopper Size
Source: Insects. 2020 Mar 10;11(3):176. doi: 10.3390/insects11030176 (PMC7142927; doi:10.3390/insects11030176)

Fig S1. Unigene length distribution of transcriptome assembly in *Romalea microptera*


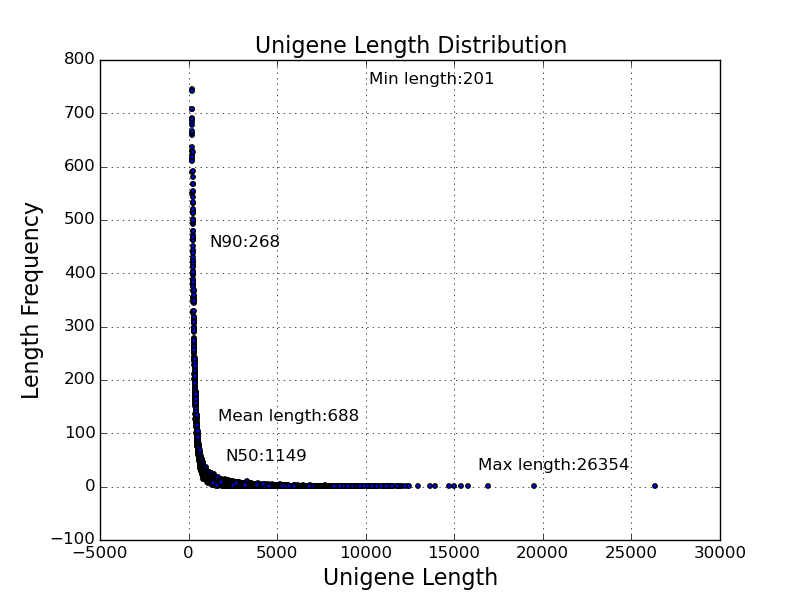

Supplement: Supplementary file 1 [file insects-11-00176-s001.zip › Supplementary Materials/Fig S1 Unigene length distribution of transcriptom.docx]
